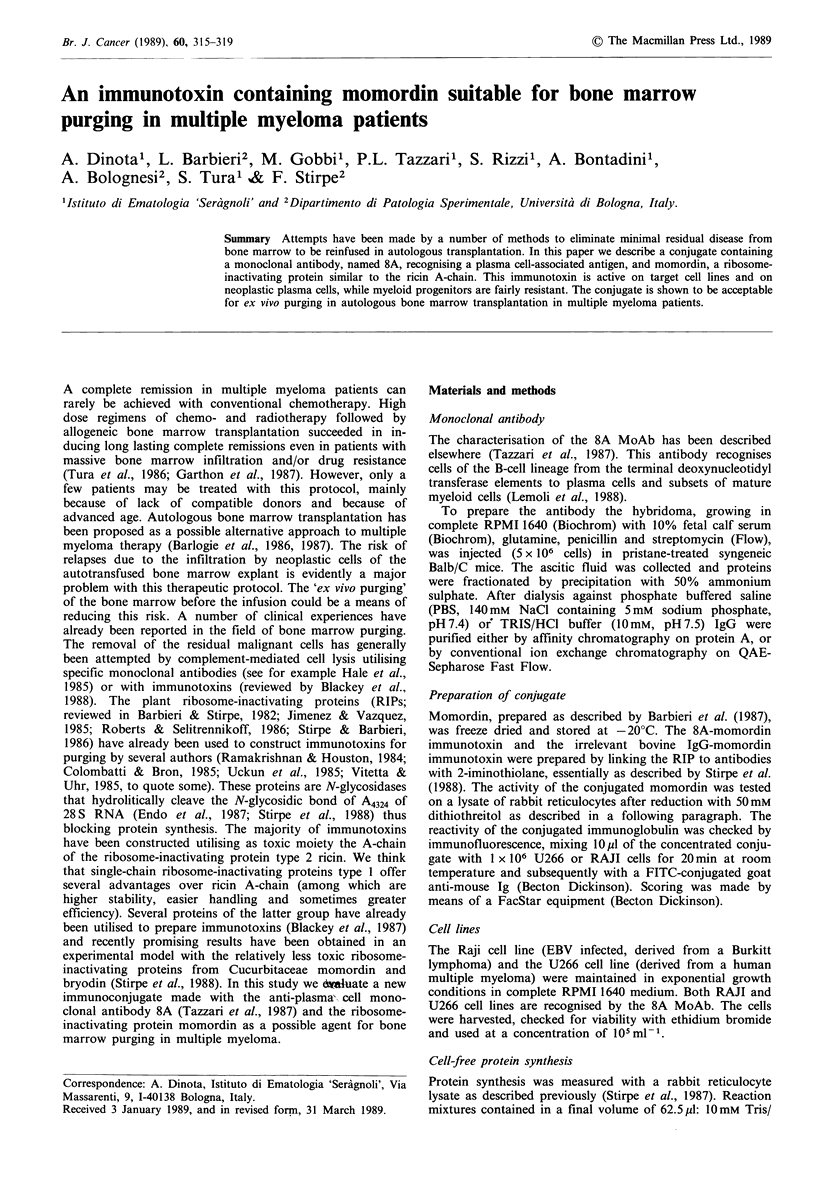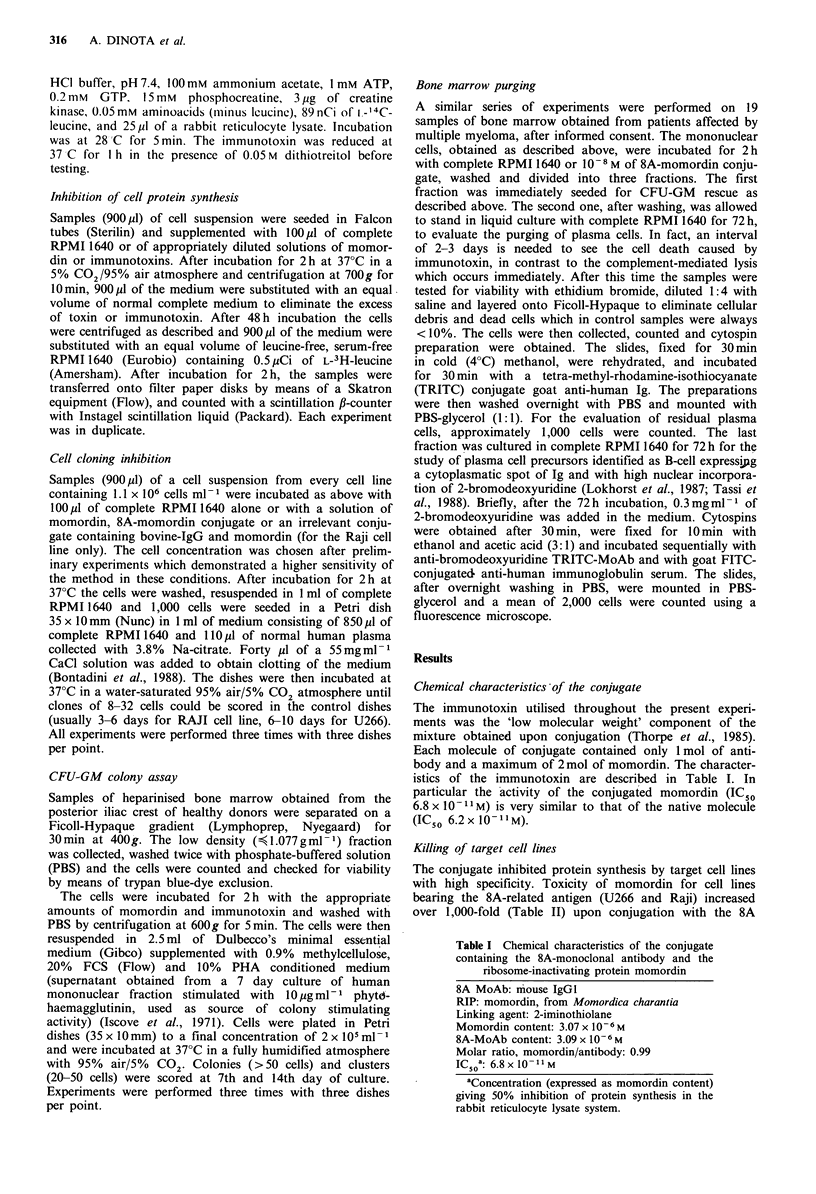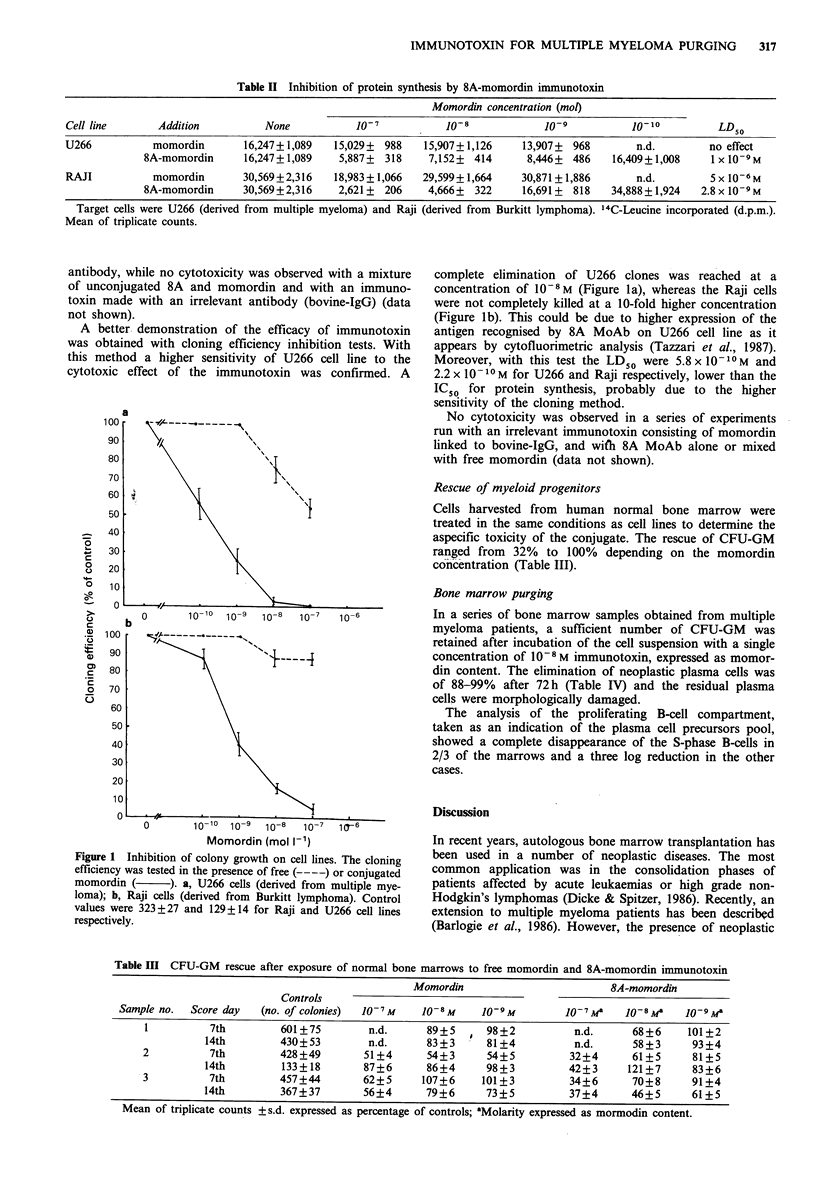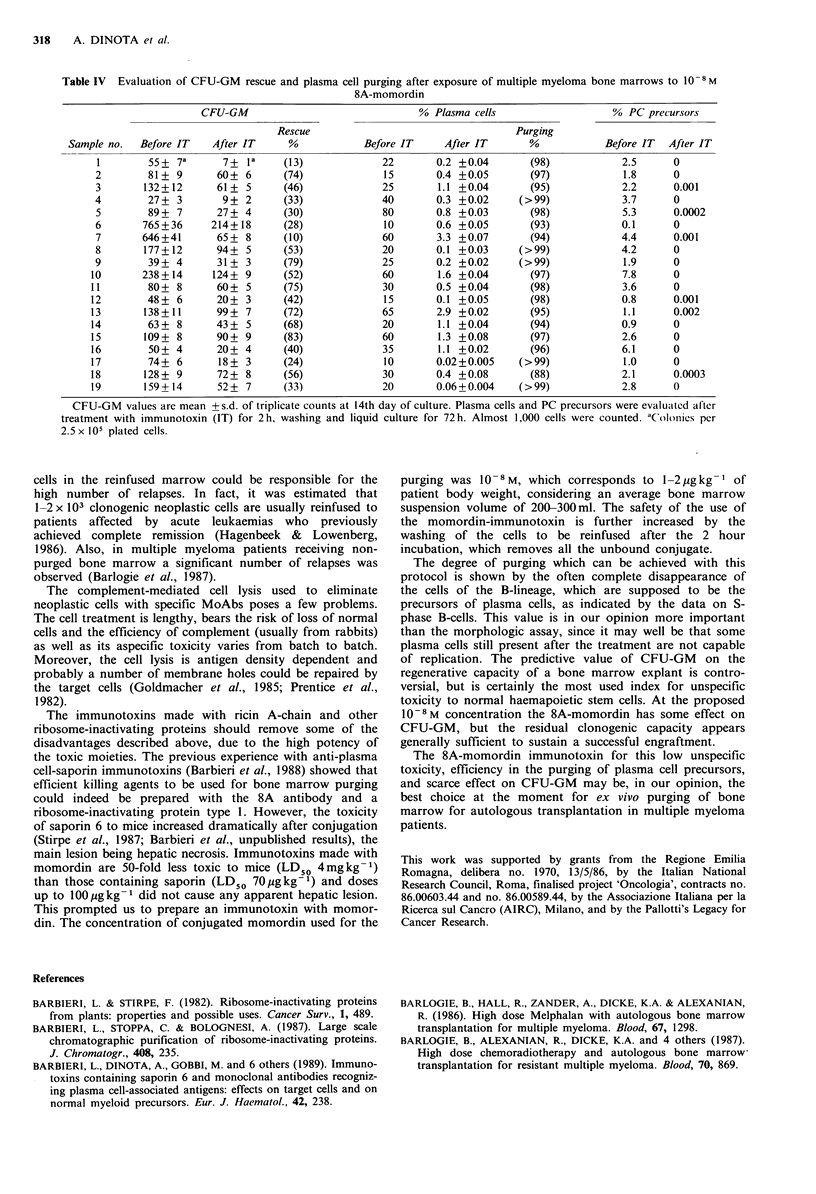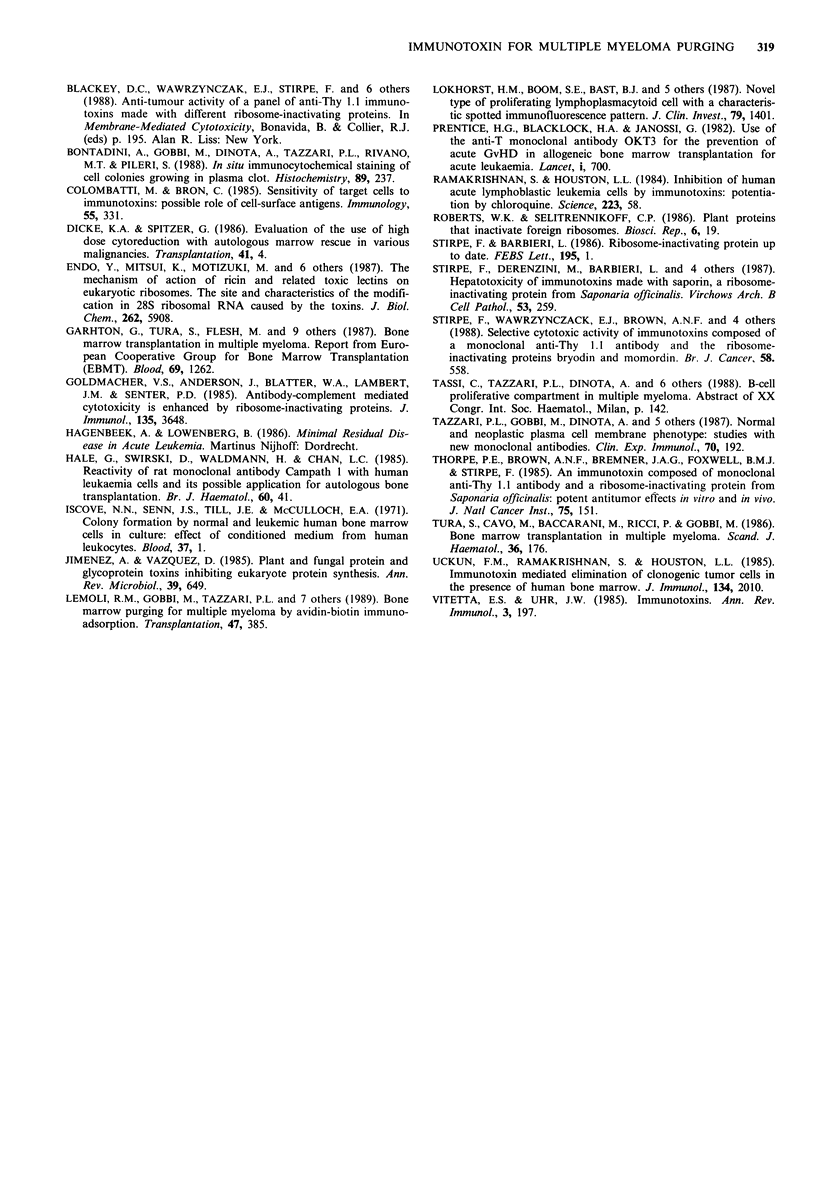# An immunotoxin containing momordin suitable for bone marrow purging in multiple myeloma patients.

**DOI:** 10.1038/bjc.1989.277

**Published:** 1989-09

**Authors:** A. Dinota, L. Barbieri, M. Gobbi, P. L. Tazzari, S. Rizzi, A. Bontadini, A. Bolognesi, S. Tura, F. Stirpe

**Affiliations:** Istituto di Ematologia SerÃ gnoli, UniversitÃ di Bologna, Italy.

## Abstract

Attempts have been made by a number of methods to eliminate minimal residual disease from bone marrow to be reinfused in autologous transplantation. In this paper we describe a conjugate containing a monoclonal antibody, named 8A, recognising a plasma cell-associated antigen, and momordin, a ribosome-inactivating protein similar to the ricin A-chain. This immunotoxin is active on target cell lines and on neoplastic plasma cells, while myeloid progenitors are fairly resistant. The conjugate is shown to be acceptable for ex vivo purging in autologous bone marrow transplantation in multiple myeloma patients.


Br. J. Cancer (1989), 60, 315–319 © The Macmillan Press Ltd., 1989

### An immunotoxin containing momordin suitable for bone marrow purging in multiple myeloma patients

A. Dinota[1], L. Barbieri[2], M. Gobbi[1], P.L. Tazzari[1], S. Rizzi[1], A. Bontadini[1], A. Bolognesi[2], S. Tura[1] & F. Stirpe[2]

[1]*Istituto di Ematologia 'Seràgnoli' and* [2]*Dipartimento di Patologia Sperimentale, Università di Bologna, Italy.*

**Summary** Attempts have been made by a number of methods to eliminate minimal residual disease from bone marrow to be reinfused in autologous transplantation. In this paper we describe a conjugate containing a monoclonal antibody, named 8A, recognising a plasma cell-associated antigen, and momordin, a ribosome-inactivating protein similar to the ricin A-chain. This immunotoxin is active on target cell lines and on neoplastic plasma cells, while myeloid progenitors are fairly resistant. The conjugate is shown to be acceptable for *ex vivo* purging in autologous bone marrow transplantation in multiple myeloma patients.

A complete remission in multiple myeloma patients can rarely be achieved with conventional chemotherapy. High dose regimens of chemo- and radiotherapy followed by allogeneic bone marrow transplantation succeeded in inducing long lasting complete remissions even in patients with massive bone marrow infiltration and/or drug resistance (Tura *et al.*, 1986; Garthon *et al.*, 1987). However, only a few patients may be treated with this protocol, mainly because of lack of compatible donors and because of advanced age. Autologous bone marrow transplantation has been proposed as a possible alternative approach to multiple myeloma therapy (Barlogie *et al.*, 1986, 1987). The risk of relapses due to the infiltration by neoplastic cells of the autotransfused bone marrow explant is evidently a major problem with this therapeutic protocol. The '*ex vivo* purging' of the bone marrow before the infusion could be a means of reducing this risk. A number of clinical experiences have already been reported in the field of bone marrow purging. The removal of the residual malignant cells has generally been attempted by complement-mediated cell lysis utilising specific monoclonal antibodies (see for example Hale *et al.*, 1985) or with immunotoxins (reviewed by Blackey *et al.*, 1988). The plant ribosome-inactivating proteins (RIPs; reviewed in Barbieri & Stirpe, 1982; Jimenez & Vazquez, 1985; Roberts & Selitrennikoff, 1986; Stirpe & Barbieri, 1986) have already been used to construct immunotoxins for purging by several authors (Ramakrishnan & Houston, 1984; Colombatti & Bron, 1985; Uckun *et al.*, 1985; Vitetta & Uhr, 1985, to quote some). These proteins are *N*-glycosidases that hydrolitically cleave the *N*-glycosidic bond of $A_{4324}$ of 28S RNA (Endo *et al.*, 1987; Stirpe *et al.*, 1988) thus blocking protein synthesis. The majority of immunotoxins have been constructed utilising as toxic moiety the A-chain of the ribosome-inactivating protein type 2 ricin. We think that single-chain ribosome-inactivating proteins type 1 offer several advantages over ricin A-chain (among which are higher stability, easier handling and sometimes greater efficiency). Several proteins of the latter group have already been utilised to prepare immunotoxins (Blackey *et al.*, 1987) and recently promising results have been obtained in an experimental model with the relatively less toxic ribosome-inactivating proteins from Cucurbitaceae momordin and bryodin (Stirpe *et al.*, 1988). In this study we evaluate a new immunoconjugate made with the anti-plasma cell monoclonal antibody 8A (Tazzari *et al.*, 1987) and the ribosome-inactivating protein momordin as a possible agent for bone marrow purging in multiple myeloma.

Correspondence: A. Dinota, Istituto di Ematologia 'Seràgnoli', Via Massarenti, 9, I-40138 Bologna, Italy.
Received 3 January 1989, and in revised form, 31 March 1989.

### Materials and methods

### *Monoclonal antibody*

The characterisation of the 8A MoAb has been described elsewhere (Tazzari *et al.*, 1987). This antibody recognises cells of the B-cell lineage from the terminal deoxynucleotidyl transferase elements to plasma cells and subsets of mature myeloid cells (Lemoli *et al.*, 1988).

To prepare the antibody the hybridoma, growing in complete RPMI 1640 (Biochrom) with 10% fetal calf serum (Biochrom), glutamine, penicillin and streptomycin (Flow), was injected ($5 \times 10^6$ cells) in pristane-treated syngeneic Balb/C mice. The ascitic fluid was collected and proteins were fractionated by precipitation with 50% ammonium sulphate. After dialysis against phosphate buffered saline (PBS, 140 mM NaCl containing 5 mM sodium phosphate, pH 7.4) or TRIS/HCl buffer (10 mM, pH 7.5) IgG were purified either by affinity chromatography on protein A, or by conventional ion exchange chromatography on QAE-Sepharose Fast Flow.

### *Preparation of conjugate*

Momordin, prepared as described by Barbieri *et al.* (1987), was freeze dried and stored at −20°C. The 8A-momordin immunotoxin and the irrelevant bovine IgG-momordin immunotoxin were prepared by linking the RIP to antibodies with 2-iminothiolane, essentially as described by Stirpe *et al.* (1988). The activity of the conjugated momordin was tested on a lysate of rabbit reticulocytes after reduction with 50 mM dithiothreitol as described in a following paragraph. The reactivity of the conjugated immunoglobulin was checked by immunofluorescence, mixing 10 $\mu$l of the concentrated conjugate with $1 \times 10^6$ U266 or RAJI cells for 20 min at room temperature and subsequently with a FITC-conjugated goat anti-mouse Ig (Becton Dickinson). Scoring was made by means of a FacStar equipment (Becton Dickinson).

### *Cell lines*

The Raji cell line (EBV infected, derived from a Burkitt lymphoma) and the U266 cell line (derived from a human multiple myeloma) were maintained in exponential growth conditions in complete RPMI 1640 medium. Both RAJI and U266 cell lines are recognised by the 8A MoAb. The cells were harvested, checked for viability with ethidium bromide and used at a concentration of $10^5$ ml$^{-1}$.

### *Cell-free protein synthesis*

Protein synthesis was measured with a rabbit reticulocyte lysate as described previously (Stirpe *et al.*, 1987). Reaction mixtures contained in a final volume of 62.5 $\mu$l: 10 mM Tris/

HCl buffer, pH 7.4, 100 mM ammonium acetate, 1 mM ATP, 0.2 mM GTP, 15 mM phosphocreatine, 3 $\mu$g of creatine kinase, 0.05 mM aminoacids (minus leucine), 89 nCi of L-$^{14}$C-leucine, and 25 $\mu$l of a rabbit reticulocyte lysate. Incubation was at 28°C for 5 min. The immunotoxin was reduced at 37°C for 1 h in the presence of 0.05 M dithiotreitol before testing.

*Inhibition of cell protein synthesis*

Samples (900 $\mu$l) of cell suspension were seeded in Falcon tubes (Sterilin) and supplemented with 100 $\mu$l of complete RPMI 1640 or of appropriately diluted solutions of momordin or immunotoxins. After incubation for 2 h at 37°C in a 5% $CO_2$/95% air atmosphere and centrifugation at 700 $g$ for 10 min, 900 $\mu$l of the medium were substituted with an equal volume of normal complete medium to eliminate the excess of toxin or immunotoxin. After 48 h incubation the cells were centrifuged as described and 900 $\mu$l of the medium were substituted with an equal volume of leucine-free, serum-free RPMI 1640 (Eurobio) containing 0.5 $\mu$Ci of L-$^{3}$H-leucine (Amersham). After incubation for 2 h, the samples were transferred onto filter paper disks by means of a Skatron equipment (Flow), and counted with a scintillation $\beta$-counter with Instagel scintillation liquid (Packard). Each experiment was in duplicate.

*Cell cloning inhibition*

Samples (900 $\mu$l) of a cell suspension from every cell line containing $1.1 \times 10^6$ cells ml$^{-1}$ were incubated as above with 100 $\mu$l of complete RPMI 1640 alone or with a solution of momordin, 8A-momordin conjugate or an irrelevant conjugate containing bovine-IgG and momordin (for the Raji cell line only). The cell concentration was chosen after preliminary experiments which demonstrated a higher sensitivity of the method in these conditions. After incubation for 2 h at 37°C the cells were washed, resuspended in 1 ml of complete RPMI 1640 and 1,000 cells were seeded in a Petri dish 35 × 10 mm (Nunc) in 1 ml of medium consisting of 850 $\mu$l of complete RPMI 1640 and 110 $\mu$l of normal human plasma collected with 3.8% Na-citrate. Forty $\mu$l of a 55 mg ml$^{-1}$ CaCl solution was added to obtain clotting of the medium (Bontadini *et al.*, 1988). The dishes were then incubated at 37°C in a water-saturated 95% air/5% $CO_2$ atmosphere until clones of 8–32 cells could be scored in the control dishes (usually 3–6 days for RAJI cell line, 6–10 days for U266). All experiments were performed three times with three dishes per point.

*CFU-GM colony assay*

Samples of heparinised bone marrow obtained from the posterior iliac crest of healthy donors were separated on a Ficoll-Hypaque gradient (Lymphoprep, Nyegaard) for 30 min at 400 $g$. The low density ($\leqslant$1.077 g ml$^{-1}$) fraction was collected, washed twice with phosphate-buffered solution (PBS) and the cells were counted and checked for viability by means of trypan blue-dye exclusion.

The cells were incubated for 2 h with the appropriate amounts of momordin and immunotoxin and washed with PBS by centrifugation at 600 $g$ for 5 min. The cells were then resuspended in 2.5 ml of Dulbecco's minimal essential medium (Gibco) supplemented with 0.9% methylcellulose, 20% FCS (Flow) and 10% PHA conditioned medium (supernatant obtained from a 7 day culture of human mononuclear fraction stimulated with 10 $\mu$g ml$^{-1}$ phytohaemagglutinin, used as source of colony stimulating activity) (Iscove *et al.*, 1971). Cells were plated in Petri dishes (35 × 10 mm) to a final concentration of $2 \times 10^5$ ml$^{-1}$ and were incubated at 37°C in a fully humidified atmosphere with 95% air/5% $CO_2$. Colonies (>50 cells) and clusters (20–50 cells) were scored at 7th and 14th day of culture. Experiments were performed three times with three dishes per point.

*Bone marrow purging*

A similar series of experiments were performed on 19 samples of bone marrow obtained from patients affected by multiple myeloma, after informed consent. The mononuclear cells, obtained as described above, were incubated for 2 h with complete RPMI 1640 or $10^{-8}$ M of 8A-momordin conjugate, washed and divided into three fractions. The first fraction was immediately seeded for CFU-GM rescue as described above. The second one, after washing, was allowed to stand in liquid culture with complete RPMI 1640 for 72 h, to evaluate the purging of plasma cells. In fact, an interval of 2–3 days is needed to see the cell death caused by immunotoxin, in contrast to the complement-mediated lysis which occurs immediately. After this time the samples were tested for viability with ethidium bromide, diluted 1:4 with saline and layered onto Ficoll-Hypaque to eliminate cellular debris and dead cells which in control samples were always <10%. The cells were then collected, counted and cytospin preparation were obtained. The slides, fixed for 30 min in cold (4°C) methanol, were rehydrated, and incubated for 30 min with a tetra-methyl-rhodamine-isothiocyanate (TRITC) conjugate goat anti-human Ig. The preparations were then washed overnight with PBS and mounted with PBS-glycerol (1:1). For the evaluation of residual plasma cells, approximately 1,000 cells were counted. The last fraction was cultured in complete RPMI 1640 for 72 h for the study of plasma cell precursors identified as B-cell expressing a cytoplasmatic spot of Ig and with high nuclear incorporation of 2-bromodeoxyuridine (Lokhorst *et al.*, 1987; Tassi *et al.*, 1988). Briefly, after the 72 h incubation, 0.3 mg ml$^{-1}$ of 2-bromodeoxyuridine was added in the medium. Cytospins were obtained after 30 min, were fixed for 10 min with ethanol and acetic acid (3:1) and incubated sequentially with anti-bromodeoxyuridine TRITC-MoAb and with goat FITC-conjugated anti-human immunoglobulin serum. The slides, after overnight washing in PBS, were mounted in PBS-glycerol and a mean of 2,000 cells were counted using a fluorescence microscope.

### Results

*Chemical characteristics of the conjugate*

The immunotoxin utilised throughout the present experiments was the 'low molecular weight' component of the mixture obtained upon conjugation (Thorpe *et al.*, 1985). Each molecule of conjugate contained only 1 mol of antibody and a maximum of 2 mol of momordin. The characteristics of the immunotoxin are described in Table I. In particular the activity of the conjugated momordin ($IC_{50}$ $6.8 \times 10^{-11}$ M) is very similar to that of the native molecule ($IC_{50}$ $6.2 \times 10^{-11}$ M).

*Killing of target cell lines*

The conjugate inhibited protein synthesis by target cell lines with high specificity. Toxicity of momordin for cell lines bearing the 8A-related antigen (U266 and Raji) increased over 1,000-fold (Table II) upon conjugation with the 8A

**Table I** Chemical characteristics of the conjugate containing the 8A-monoclonal antibody and the ribosome-inactivating protein momordin

| |
|---|
| 8A MoAb: mouse IgG1 |
| RIP: momordin, from *Momordica charantia* |
| Linking agent: 2-iminothiolane |
| Momordin content: $3.07 \times 10^{-6}$ M |
| 8A-MoAb content: $3.09 \times 10^{-6}$ M |
| Molar ratio, momordin/antibody: 0.99 |
| $IC_{50}$[a]: $6.8 \times 10^{-11}$ M |

[a]Concentration (expressed as momordin content) giving 50% inhibition of protein synthesis in the rabbit reticulocyte lysate system.

**Table II** Inhibition of protein synthesis by 8A-momordin immunotoxin

| Cell line | Addition | None | *Momordin concentration (mol)* $10^{-7}$ | $10^{-8}$ | $10^{-9}$ | $10^{-10}$ | $LD_{50}$ |
|---|---|---|---|---|---|---|---|
| U266 | momordin | 16,247±1,089 | 15,029± 988 | 15,907±1,126 | 13,907± 968 | n.d. | no effect |
| | 8A-momordin | 16,247±1,089 | 5,887± 318 | 7,152± 414 | 8,446± 486 | 16,409±1,008 | $1\times10^{-9}$ M |
| RAJI | momordin | 30,569±2,316 | 18,983±1,066 | 29,599±1,664 | 30,871±1,886 | n.d. | $5\times10^{-6}$ M |
| | 8A-momordin | 30,569±2,316 | 2,621± 206 | 4,666± 322 | 16,691± 818 | 34,888±1,924 | $2.8\times10^{-9}$ M |

Target cells were U266 (derived from multiple myeloma) and Raji (derived from Burkitt lymphoma). $^{14}$C-Leucine incorporated (d.p.m.). Mean of triplicate counts.

antibody, while no cytotoxicity was observed with a mixture of unconjugated 8A and momordin and with an immunotoxin made with an irrelevant antibody (bovine-IgG) (data not shown).

A better demonstration of the efficacy of immunotoxin was obtained with cloning efficiency inhibition tests. With this method a higher sensitivity of U266 cell line to the cytotoxic effect of the immunotoxin was confirmed. A complete elimination of U266 clones was reached at a concentration of $10^{-8}$ M (Figure 1a), whereas the Raji cells were not completely killed at a 10-fold higher concentration (Figure 1b). This could be due to higher expression of the antigen recognised by 8A MoAb on U266 cell line as it appears by cytofluorimetric analysis (Tazzari *et al.*, 1987). Moreover, with this test the $LD_{50}$ were $5.8\times10^{-10}$ M and $2.2\times10^{-10}$ M for U266 and Raji respectively, lower than the $IC_{50}$ for protein synthesis, probably due to the higher sensitivity of the cloning method.

No cytotoxicity was observed in a series of experiments run with an irrelevant immunotoxin consisting of momordin linked to bovine-IgG, and with 8A MoAb alone or mixed with free momordin (data not shown).

**Figure 1** Inhibition of colony growth on cell lines. The cloning efficiency was tested in the presence of free (----) or conjugated momordin (——). **a**, U266 cells (derived from multiple myeloma); **b**, Raji cells (derived from Burkitt lymphoma). Control values were 323±27 and 129±14 for Raji and U266 cell lines respectively.

### *Rescue of myeloid progenitors*

Cells harvested from human normal bone marrow were treated in the same conditions as cell lines to determine the aspecific toxicity of the conjugate. The rescue of CFU-GM ranged from 32% to 100% depending on the momordin concentration (Table III).

### *Bone marrow purging*

In a series of bone marrow samples obtained from multiple myeloma patients, a sufficient number of CFU-GM was retained after incubation of the cell suspension with a single concentration of $10^{-8}$ M immunotoxin, expressed as momordin content. The elimination of neoplastic plasma cells was of 88–99% after 72 h (Table IV) and the residual plasma cells were morphologically damaged.

The analysis of the proliferating B-cell compartment, taken as an indication of the plasma cell precursors pool, showed a complete disappearance of the S-phase B-cells in 2/3 of the marrows and a three log reduction in the other cases.

### Discussion

In recent years, autologous bone marrow transplantation has been used in a number of neoplastic diseases. The most common application was in the consolidation phases of patients affected by acute leukaemias or high grade non-Hodgkin's lymphomas (Dicke & Spitzer, 1986). Recently, an extension to multiple myeloma patients has been described (Barlogie *et al.*, 1986). However, the presence of neoplastic

**Table III** CFU-GM rescue after exposure of normal bone marrows to free momordin and 8A-momordin immunotoxin

| Sample no. | Score day | Controls (no. of colonies) | *Momordin* $10^{-7}$ M | $10^{-8}$ M | $10^{-9}$ M | *8A-momordin* $10^{-7}$ M[a] | $10^{-8}$ M[a] | $10^{-9}$ M[a] |
|---|---|---|---|---|---|---|---|---|
| 1 | 7th | 601±75 | n.d. | 89±5 | 98±2 | n.d. | 68±6 | 101±2 |
| | 14th | 430±53 | n.d. | 83±3 | 81±4 | n.d. | 58±3 | 93±4 |
| 2 | 7th | 428±49 | 51±4 | 54±3 | 54±5 | 32±4 | 61±5 | 81±5 |
| | 14th | 133±18 | 87±6 | 86±4 | 98±3 | 42±3 | 121±7 | 83±6 |
| 3 | 7th | 457±44 | 62±5 | 107±6 | 101±3 | 34±6 | 70±8 | 91±4 |
| | 14th | 367±37 | 56±4 | 79±6 | 73±5 | 37±4 | 46±5 | 61±5 |

Mean of triplicate counts ±s.d. expressed as percentage of controls; [a]Molarity expressed as mormodin content.

**Table IV** Evaluation of CFU-GM rescue and plasma cell purging after exposure of multiple myeloma bone marrows to $10^{-8}$ M 8A-momordin

| Sample no. | CFU-GM Before IT | CFU-GM After IT | CFU-GM Rescue % | % Plasma cells Before IT | % Plasma cells After IT | % Plasma cells Purging % | % PC precursors Before IT | % PC precursors After IT |
|---|---|---|---|---|---|---|---|---|
| 1 | 55± 7[a] | 7± 1[a] | (13) | 22 | 0.2 ±0.04 | (98) | 2.5 | 0 |
| 2 | 81± 9 | 60± 6 | (74) | 15 | 0.4 ±0.05 | (97) | 1.8 | 0 |
| 3 | 132±12 | 61± 5 | (46) | 25 | 1.1 ±0.04 | (95) | 2.2 | 0.001 |
| 4 | 27± 3 | 9± 2 | (33) | 40 | 0.3 ±0.02 | (>99) | 3.7 | 0 |
| 5 | 89± 7 | 27± 4 | (30) | 80 | 0.8 ±0.03 | (98) | 5.3 | 0.0002 |
| 6 | 765±36 | 214±18 | (28) | 10 | 0.6 ±0.05 | (93) | 0.1 | 0 |
| 7 | 646±41 | 65± 8 | (10) | 60 | 3.3 ±0.07 | (94) | 4.4 | 0.001 |
| 8 | 177±12 | 94± 5 | (53) | 20 | 0.1 ±0.03 | (>99) | 4.2 | 0 |
| 9 | 39± 4 | 31± 3 | (79) | 25 | 0.2 ±0.02 | (>99) | 1.9 | 0 |
| 10 | 238±14 | 124± 9 | (52) | 60 | 1.6 ±0.04 | (97) | 7.8 | 0 |
| 11 | 80± 8 | 60± 5 | (75) | 30 | 0.5 ±0.04 | (98) | 3.6 | 0 |
| 12 | 48± 6 | 20± 3 | (42) | 15 | 0.1 ±0.05 | (98) | 0.8 | 0.001 |
| 13 | 138±11 | 99± 7 | (72) | 65 | 2.9 ±0.02 | (95) | 1.1 | 0.002 |
| 14 | 63± 8 | 43± 5 | (68) | 20 | 1.1 ±0.04 | (94) | 0.9 | 0 |
| 15 | 109± 8 | 90± 9 | (83) | 60 | 1.3 ±0.08 | (97) | 2.6 | 0 |
| 16 | 50± 4 | 20± 4 | (40) | 35 | 1.1 ±0.02 | (96) | 6.1 | 0 |
| 17 | 74± 6 | 18± 3 | (24) | 10 | 0.02±0.005 | (>99) | 1.0 | 0 |
| 18 | 128± 9 | 72± 8 | (56) | 30 | 0.4 ±0.08 | (88) | 2.1 | 0.0003 |
| 19 | 159±14 | 52± 7 | (33) | 20 | 0.06±0.004 | (>99) | 2.8 | 0 |

CFU-GM values are mean ±s.d. of triplicate counts at 14th day of culture. Plasma cells and PC precursors were evaluated after treatment with immunotoxin (IT) for 2 h, washing and liquid culture for 72 h. Almost 1,000 cells were counted. [a]Colonies per $2.5\times10^5$ plated cells.

cells in the reinfused marrow could be responsible for the high number of relapses. In fact, it was estimated that $1–2\times10^3$ clonogenic neoplastic cells are usually reinfused to patients affected by acute leukaemias who previously achieved complete remission (Hagenbeek & Lowenberg, 1986). Also, in multiple myeloma patients receiving non-purged bone marrow a significant number of relapses was observed (Barlogie *et al.*, 1987).

The complement-mediated cell lysis used to eliminate neoplastic cells with specific MoAbs poses a few problems. The cell treatment is lengthy, bears the risk of loss of normal cells and the efficiency of complement (usually from rabbits) as well as its aspecific toxicity varies from batch to batch. Moreover, the cell lysis is antigen density dependent and probably a number of membrane holes could be repaired by the target cells (Goldmacher *et al.*, 1985; Prentice *et al.*, 1982).

The immunotoxins made with ricin A-chain and other ribosome-inactivating proteins should remove some of the disadvantages described above, due to the high potency of the toxic moieties. The previous experience with anti-plasma cell-saporin immunotoxins (Barbieri *et al.*, 1988) showed that efficient killing agents to be used for bone marrow purging could indeed be prepared with the 8A antibody and a ribosome-inactivating protein type 1. However, the toxicity of saporin 6 to mice increased dramatically after conjugation (Stirpe *et al.*, 1987; Barbieri *et al.*, unpublished results), the main lesion being hepatic necrosis. Immunotoxins made with momordin are 50-fold less toxic to mice ($LD_{50}$ 4 mg kg$^{-1}$) than those containing saporin ($LD_{50}$ 70 μg kg$^{-1}$) and doses up to 100 μg kg$^{-1}$ did not cause any apparent hepatic lesion. This prompted us to prepare an immunotoxin with momordin. The concentration of conjugated momordin used for the purging was $10^{-8}$ M, which corresponds to 1–2 μg kg$^{-1}$ of patient body weight, considering an average bone marrow suspension volume of 200–300 ml. The safety of the use of the momordin-immunotoxin is further increased by the washing of the cells to be reinfused after the 2 hour incubation, which removes all the unbound conjugate.

The degree of purging which can be achieved with this protocol is shown by the often complete disappearance of the cells of the B-lineage, which are supposed to be the precursors of plasma cells, as indicated by the data on S-phase B-cells. This value is in our opinion more important than the morphologic assay, since it may well be that some plasma cells still present after the treatment are not capable of replication. The predictive value of CFU-GM on the regenerative capacity of a bone marrow explant is controversial, but is certainly the most used index for unspecific toxicity to normal haemapoietic stem cells. At the proposed $10^{-8}$ M concentration the 8A-momordin has some effect on CFU-GM, but the residual clonogenic capacity appears generally sufficient to sustain a successful engraftment.

The 8A-momordin immunotoxin for this low unspecific toxicity, efficiency in the purging of plasma cell precursors, and scarce effect on CFU-GM may be, in our opinion, the best choice at the moment for *ex vivo* purging of bone marrow for autologous transplantation in multiple myeloma patients.

This work was supported by grants from the Regione Emilia Romagna, delibera no. 1970, 13/5/86, by the Italian National Research Council, Roma, finalised project 'Oncologia', contracts no. 86.00603.44 and no. 86.00589.44, by the Associazione Italiana per la Ricerca sul Cancro (AIRC), Milano, and by the Pallotti's Legacy for Cancer Research.

### References

BARBIERI, L. & STIRPE, F. (1982). Ribosome-inactivating proteins from plants: properties and possible uses. *Cancer Surv.*, **1**, 489.

BARBIERI, L., STOPPA, C. & BOLOGNESI, A. (1987). Large scale chromatographic purification of ribosome-inactivating proteins. *J. Chromatogr.*, **408**, 235.

BARBIERI, L., DINOTA, A., GOBBI, M. and 6 others (1989). Immunotoxins containing saporin 6 and monoclonal antibodies recognizing plasma cell-associated antigens: effects on target cells and on normal myeloid precursors. *Eur. J. Haematol.*, **42**, 238.

BARLOGIE, B., HALL, R., ZANDER, A., DICKE, K.A. & ALEXANIAN, R. (1986). High dose Melphalan with autologous bone marrow transplantation for multiple myeloma. *Blood*, **67**, 1298.

BARLOGIE, B., ALEXANIAN, R., DICKE, K.A. and 4 others (1987). High dose chemoradiotherapy and autologous bone marrow transplantation for resistant multiple myeloma. *Blood*, **70**, 869.

BLACKEY, D.C., WAWRZYNCZAK, E.J., STIRPE, F. and 6 others (1988). Anti-tumour activity of a panel of anti-Thy 1.1 immunotoxins made with different ribosome-inactivating proteins. In *Membrane-Mediated Cytotoxicity*, Bonavida, B. & Collier, R.J. (eds) p. 195. Alan R. Liss: New York.

BONTADINI, A., GOBBI, M., DINOTA, A., TAZZARI, P.L., RIVANO, M.T. & PILERI, S. (1988). *In situ* immunocytochemical staining of cell colonies growing in plasma clot. *Histochemistry*, **89**, 237.

COLOMBATTI, M. & BRON, C. (1985). Sensitivity of target cells to immunotoxins: possible role of cell-surface antigens. *Immunology*, **55**, 331.

DICKE, K.A. & SPITZER, G. (1986). Evaluation of the use of high dose cytoreduction with autologous marrow rescue in various malignancies. *Transplantation*, **41**, 4.

ENDO, Y., MITSUI, K., MOTIZUKI, M. and 6 others (1987). The mechanism of action of ricin and related toxic lectins on eukaryotic ribosomes. The site and characteristics of the modification in 28S ribosomal RNA caused by the toxins. *J. Biol. Chem.*, **262**, 5908.

GARHTON, G., TURA, S., FLESH, M. and 9 others (1987). Bone marrow transplantation in multiple myeloma. Report from European Cooperative Group for Bone Marrow Transplantation (EBMT). *Blood*, **69**, 1262.

GOLDMACHER, V.S., ANDERSON, J., BLATTER, W.A., LAMBERT, J.M. & SENTER, P.D. (1985). Antibody-complement mediated cytotoxicity is enhanced by ribosome-inactivating proteins. *J. Immunol.*, **135**, 3648.

HAGENBEEK, A. & LOWENBERG, B. (1986). *Minimal Residual Disease in Acute Leukemia.* Martinus Nijhoff: Dordrecht.

HALE, G., SWIRSKI, D., WALDMANN, H. & CHAN, L.C. (1985). Reactivity of rat monoclonal antibody Campath 1 with human leukaemia cells and its possible application for autologous bone transplantation. *Br. J. Haematol.*, **60**, 41.

ISCOVE, N.N., SENN, J.S., TILL, J.E. & McCULLOCH, E.A. (1971). Colony formation by normal and leukemic human bone marrow cells in culture: effect of conditioned medium from human leukocytes. *Blood*, **37**, 1.

JIMENEZ, A. & VAZQUEZ, D. (1985). Plant and fungal protein and glycoprotein toxins inhibiting eukaryote protein synthesis. *Ann. Rev. Microbiol.*, **39**, 649.

LEMOLI, R.M., GOBBI, M., TAZZARI, P.L. and 7 others (1989). Bone marrow purging for multiple myeloma by avidin-biotin immunoadsorption. *Transplantation*, **47**, 385.

LOKHORST, H.M., BOOM, S.E., BAST, B.J. and 5 others (1987). Novel type of proliferating lymphoplasmacytoid cell with a characteristic spotted immunofluorescence pattern. *J. Clin. Invest.*, **79**, 1401.

PRENTICE, H.G., BLACKLOCK, H.A. & JANOSSI, G. (1982). Use of the anti-T monoclonal antibody OKT3 for the prevention of acute GvHD in allogeneic bone marrow transplantation for acute leukaemia. *Lancet*, **i**, 700.

RAMAKRISHNAN, S. & HOUSTON, L.L. (1984). Inhibition of human acute lymphoblastic leukemia cells by immunotoxins: potentiation by chloroquine. *Science*, **223**, 58.

ROBERTS, W.K. & SELITRENNIKOFF, C.P. (1986). Plant proteins that inactivate foreign ribosomes. *Biosci. Rep.*, **6**, 19.

STIRPE, F. & BARBIERI, L. (1986). Ribosome-inactivating protein up to date. *FEBS Lett.*, **195**, 1.

STIRPE, F., DERENZINI, M., BARBIERI, L. and 4 others (1987). Hepatotoxicity of immunotoxins made with saporin, a ribosome-inactivating protein from *Saponaria officinalis*. *Virchows Arch. B Cell Pathol.*, **53**, 259.

STIRPE, F., WAWRZYNCZACK, E.J., BROWN, A.N.F. and 4 others (1988). Selective cytotoxic activity of immunotoxins composed of a monoclonal anti-Thy 1.1 antibody and the ribosome-inactivating proteins bryodin and momordin. *Br. J. Cancer*, **58**. 558.

TASSI, C., TAZZARI, P.L., DINOTA, A. and 6 others (1988). B-cell proliferative compartment in multiple myeloma. Abstract of XX Congr. Int. Soc. Haematol., Milan, p. 142.

TAZZARI, P.L., GOBBI, M., DINOTA, A. and 5 others (1987). Normal and neoplastic plasma cell membrane phenotype: studies with new monoclonal antibodies. *Clin. Exp. Immunol.*, **70**, 192.

THORPE, P.E., BROWN, A.N.F., BREMNER, J.A.G., FOXWELL, B.M.J. & STIRPE, F. (1985). An immunotoxin composed of monoclonal anti-Thy 1.1 antibody and a ribosome-inactivating protein from *Saponaria officinalis*: potent antitumor effects *in vitro* and *in vivo*. *J. Natl Cancer Inst.*, **75**, 151.

TURA, S., CAVO, M., BACCARANI, M., RICCI, P. & GOBBI, M. (1986). Bone marrow transplantation in multiple myeloma. *Scand. J. Haematol.*, **36**, 176.

UCKUN, F.M., RAMAKRISHNAN, S. & HOUSTON, L.L. (1985). Immunotoxin mediated elimination of clonogenic tumor cells in the presence of human bone marrow. *J. Immunol.*, **134**, 2010.

VITETTA, E.S. & UHR, J.W. (1985). Immunotoxins. *Ann. Rev. Immunol.*, **3**, 197.